# Editorial: Bidirectional Communication Between Synapses and Nucleus in Brain Physiology and Disease

**DOI:** 10.3389/fnmol.2022.909036

**Published:** 2022-05-06

**Authors:** Arnaldo Parra-Damas, Toh Hean Ch'ng, Bryen A. Jordan, Carlos A. Saura

**Affiliations:** ^1^Institut de Neurociències, Departament de Bioquímica i Biologia Molecular, Universitat Autònoma de Barcelona, Barcelona, Spain; ^2^Centro de Investigación Biomédica en Red Enfermedades Neurodegenerativas (CIBERNED), Barcelona, Spain; ^3^Lee Kong Chian School of Medicine, Nanyang Technological University, Singapore, Singapore; ^4^School of Biological Science, Nanyang Technological University, Singapore, Singapore; ^5^Dominick P. Purpura Department of Neuroscience, Albert Einstein College of Medicine, Bronx, NY, United States; ^6^Department of Psychiatry and Behavioral Sciences, Albert Einstein College of Medicine, Bronx, NY, United States

**Keywords:** synapse-to-nucleus, synaptonuclear, neurodegeneration, neuropsychiatric, neurodevelopmental, transcription factors, local translation

The brain's ability to sense, process, and respond to internal and external cues is supported by nuclear gene expression triggered from distally activated dendrites and synapses. The resulting gene products -including proteins and non-coding RNAs- in turn modulate synaptic activity and plasticity, establishing a continuous crosstalk that is essential for proper function of neurons and neural circuits (Cohen and Greenberg, 2008). Bidirectional communication between synapses and the nucleus is mediated by multiple synaptonuclear factors that have recently emerged as key regulators of brain function in physiological and pathological conditions. Recent advances in this field include: the identification of new synaptonuclear factors; the elucidation of molecular mechanisms regulating their activity, localization, and transport; the characterization of their specific biological functions; and finally their involvement in a wide range of brain disorders, including neurodevelopmental, neuropsychiatric, and neurodegenerative diseases (Parra-Damas and Saura, 2019). These studies indicate that a precise bidirectional crosstalk between pre- and post-synapses and the nucleus is essential for proper neuronal function and plasticity, whereas dysregulation of such communication may lead to brain pathologies (Figure 1).

**Figure 1:**
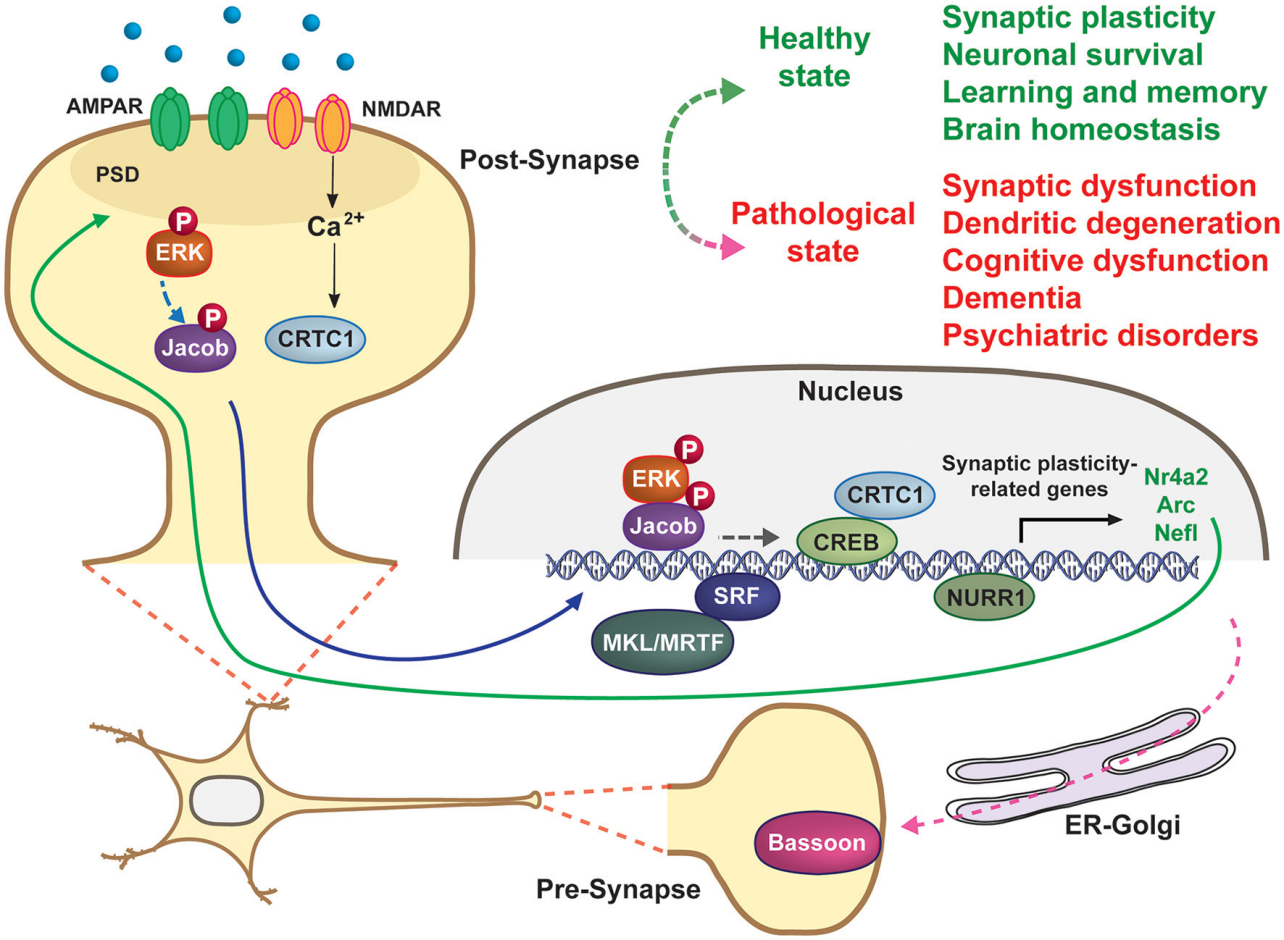
Synapse-to-nucleus signaling regulates neuronal excitability and synapse plasticity. Several synaptic and dendritic factors (CRTC1, ERK, Jacob, MKL/MRTF, NURR1,…) are activated by synaptic activity at distal neuronal dendrites, including synapses, and then translocate to the nucleus to regulate gene transcription. Bassoon is an ER-Golgi pre-synaptic protein essential for vesicle release and neurotransmission. Blue line indicates signals traveling from dendritic spines or dendrites to the nucleus to regulate gene expression, whereas the solid green line shows how gene products act at synapses to regulate synaptic plasticity and neuronal excitability. Some brain physiological and pathological processes directly or indirectly regulated by synapse-to-nucleus signaling are indicated on the top right.

This Research Topic “*Bidirectional Communication Between Synapses and Nucleus in Brain Physiology and Disease*” brings together original and review articles from 24 authors that have directly contributed to our current understanding of regulatory mechanisms underlying synapse-to-nucleus communication in physiological and pathological contexts, concerning diverse brain processes ranging from synaptic/dendritic plasticity and maternal behavior to brain diseases including cognitive dysfunction and neuropsychiatric disorders. A specific goal of this Research Topic is to promote the awareness about the wide relevance of synapse-to-nucleus communication in the Neurosciences, while hopefully encouraging new investigators to join the field.

Neuronal gene expression programs are mediated by activity-dependent transcriptional regulators, including transcription factors and coactivators, and synaptonuclear factors. Among these, the nuclear receptor 4A subfamily member 2 (Nr4a2/NURR1) is as an activity-dependent transcription factor essential for synaptic plasticity and memory (Hawk et al., 2012; Bridi and Abel, 2013). Rodríguez-Alvarez et al. showed that Nr4a2/NURR1 accumulates at post-synaptic compartments of the adult mouse hippocampus and in cultured hippocampal neurons upon synaptic activity, suggesting that Nr4a2 is a new synaptically-localized transcription factor that shuttles in and out of the nucleus and toward synapses in response to synaptic activity. Future studies should be aimed at identifying the mechanisms regulating nuclear/synapse NURR1 shuttling and its role in hippocampal synaptic plasticity and memory.

Bassoon is a major pre-synaptic scaffold protein essential for neurotransmitter release and synapse integrity, and it is also localized at the Golgi apparatus where it is thought to mediate assembly of precursor active zone structures via unclear mechanisms. By applying stimulated emission depletion (STED) super-resolution microscopy in cultured neurons expressing Bassoon mutants, Dresbach et al. identified the Bassoon structural domains mediating binding at the Golgi-apparatus membrane and the active zone components, revealing a structural arrangement characteristic of synapses. These results provide new insights on the topology and trafficking of precursor active zone components from the Golgi apparatus to synapses. The contribution of this process to the global landscape of synapse-to-nucleus signaling is an interesting topic that requires further investigation.

The megakaryoblastic leukemia (MKL)/myocardin-related transcription factor (MRTF) is a serum response factor (SRF) cofactor that is found at synapses and nucleus, where it regulates gene transcription mediating maturation of dendritic spines (Kaneda et al., 2018). Tabuchi and Ihara review the roles of the distinct MKL/MRTF isoforms in the brain including their differential contribution to SRF-regulated gene expression and dendritic and synapse morphology, and finally discuss evidence indicating their implication in neurological disorders.

The synaptonuclear factor Jacob links activation of NMDA receptors to nuclear gene expression required for synaptic plasticity in excitatory neurons. Karpova et al. provide a comprehensive review of the mechanisms mediating Jacob synapse-to-nucleus trafficking underlying synaptic plasticity. Special emphasis is given to the Jacob synaptic interactome and nuclear function, particularly its role in regulating gene transcription via the transcription factor cAMP-response element binding protein (CREB).

The CREB-regulated transcription coactivator-1 (CRTC1) is an activity-dependent synaptonuclear messenger that mediates transcription of neuroplasticity genes. CRTC1 has recently emerged as a central regulator of neuronal development, plasticity and other brain functions, whereas its dysfunction is linked with neuropsychiatric and neurodegenerative diseases (Saura and Cardinaux, 2017). In this Research Topic, Cardinaux et al. summarized recent evidence based on animal and human pathophysiological data emphasizing a role of CRTC1 in the neurobiology of mood disorders, including stress, major depression and comorbid obesity. Although the mechanisms by which CRTC1 regulates depressive behavior are not fully understood, this review highlights a pivotal role for CRTC1 in depressive-like behaviors through regulation of dopamine and serotonin release, epigenetic regulation of neuroplasticity genes, circadian rhythms and brain energy homeostasis.

Maternal behavior is modulated by innate and experience-dependent plasticity mechanisms (Schiavo et al., 2020). Shumyatsky et al. thoroughly reviewed the molecular mechanisms involved in parental care and discuss the interesting hypothesis that activity-dependent transcription and synapse-to-nucleus signaling related to synaptic plasticity may represent critical mechanisms contributing to maternal behavior, which could be highly relevant for targeting postpartum mental disorders such as postpartum depression.

Collectively, the articles published in this Research Topic provide a broad overview of different brain processes that engage in synapse-to-nucleus communication under physiological and pathological conditions (Figure 1). It is expected that the emerging interest in this topic and the application of new technological developments will deliver exciting findings in both basic and translational neuroscience in the upcoming years. Importantly, a better knowledge of the mechanisms governing synapse-to-nucleus communication could be essential for developing effective therapeutics and diagnostic tools for neurological and neuropsychiatric disorders.

## Author Contributions

AP-D and CAS drafted the manuscript. THC and BAJ edited the manuscript. All authors contributed to the article and approved the submitted version.

## Funding

CAS was supported by grants from the Ministerio de Ciencia e Innovación (FEDER funds) PID2019-106615RB-I00, Instituto de Salud Carlos III (CIBERNED CB06/05/0042), and Generalitat de Catalunya (2017 SGR749). AP-D was supported by a Juan de la Cierva-Incorporación Program fellowship (IJC2019-042468-I). THC was supported by Nanyang Assistant Professor (NAP) startup funds from Nanyang Technological University, Singapore and by Singapore Ministry of Education Academic Research Fund Tier 1 (MOE2018-T1-002-033). BAJ was supported by National Institutes of Health (R01NS118820). The funders had no role in the design, data collection and analysis or preparation of the manuscript.

## Conflict of Interest

The authors declare that the research was conducted in the absence of any commercial or financial relationships that could be construed as a potential conflict of interest.

## Publisher's Note

All claims expressed in this article are solely those of the authors and do not necessarily represent those of their affiliated organizations, or those of the publisher, the editors and the reviewers. Any product that may be evaluated in this article, or claim that may be made by its manufacturer, is not guaranteed or endorsed by the publisher.
